# Microstructure-Induced Anisotropic Optical Properties of YF_3_ Columnar Thin Films Prepared by Glancing Angle Deposition

**DOI:** 10.3390/nano10122413

**Published:** 2020-12-03

**Authors:** Yao Shan, Pian Liu, Yao Chen, Haotian Zhang, Huatian Tu, Yuxiang Zheng, Rongjun Zhang, Songyou Wang, Jing Li, Liangyao Chen

**Affiliations:** Department of Optical Science and Engineering, Key Laboratory of Micro and Nano Photonic Structures, Ministry of Education, Shanghai Engineering Research Center of Ultra-Precision Optical Manufacturing, Fudan University, Shanghai 200433, China; shanyao1754@163.com (Y.S.); 17110720027@fudan.edu.cn (P.L.); 18210720008@fudan.edu.cn (Y.C.); changzht@gmail.com (H.Z.); 16110720002@fudan.edu.cn (H.T.); rjzhang@fudan.edu.cn (R.Z.); sywang@fudan.ac.cn (S.W.); lijing@fudan.ac.cn (J.L.); lychen@fudan.ac.cn (L.C.)

**Keywords:** glancing angle deposition, yttrium fluoride, columnar thin films, anisotropic optical properties, Mueller matrix ellipsometry, microstructure

## Abstract

Yttrium fluoride (YF_3_) columnar thin films (CTFs) were fabricated by electron beam evaporation with the glancing angle deposition method. The microstructures and optical properties of YF_3_ CTFs were studied systematically. The YF_3_ films grown at different deposition angles are all amorphous. As the deposition angle increases, the columns in YF_3_ CTFs become increasingly separated and inclined, and the volume fraction of YF_3_ decreases, resulting in lower refractive indices. This phenomenon is attributed to the self-shadowing effect and limited adatom diffusion. The YF_3_ CTFs are optically biaxial anisotropic with the long axis (*c*-axis) parallel to the columns, the short axis (*b*-axis) perpendicular to the columns, and the other axis (*a*-axis) parallel to the film interface. The principal refractive index along the *b*-axis for the 82°-deposited sample is approximately 1.233 at 550 nm. For the 78°-deposited sample, the differences of principal refractive indices between the *c*-axis and the *b*-axis and between the *a*-axis and the *b*-axis reach the maximum 0.056 and 0.029, respectively. The differences of principal refractive indices were affected by both the deposition angle and the volume fraction of YF_3_.

## 1. Introduction

Microstructure materials have attracted great interest because of their high potential in a wide range of fields, such as electronics, photonics, magnetism, biomedicine, and chemistry [1]. Glancing angle deposition (GLAD) is an effective method for preparing variable Zone 1 columnar microstructures, which are defined as the structures of film material with a melting point three times higher than the substrate temperature [2,3]. By controlling the substrate tilt in multiple rotational ways, various columnar microstructures can be prepared by the GLAD method, such as columnar, spiral, c-shape, and z-shape [4]. The microstructure and the optical properties of the films fabricated by the GLAD method depend on the self-shadowing effect and the formation of columnar grains, leading to applications in optics, energy, electrocatalysts, electrochromism, thermochromism, etc. [5,6,7,8,9,10,11]. In addition, these highly oriented films with strong optical anisotropy have been used in specific applications, such as optical retardation plates, birefringent omnidirectional reflectors, and three-dimensional photonic crystals [12,13,14,15,16].

As a common and important fluoride compound, yttrium fluoride (YF_3_) has been widely used as nano-particles with efficient multicolor photoluminescence, high reflectors, thin-film interference filters, substitutes for radioactive ThF_4_, and anti-reflection coatings with other optical films ranging from the near-UV to IR [17,18,19,20,21,22,23]. These functions are available based on the nature of YF_3_, with low refractive index and low absorption, excellent transmittance from UV to IR, high hardness, and the desirable ability to match with other multilayer materials [19]. However, studies on the microstructure and the microstructure-induced anisotropic optical properties of YF_3_ columnar thin films (CTFs) fabricated by the GLAD technique are still limited. In this work, YF_3_ CTFs with various inclined angles were prepared by the GLAD technique, and their microstructures and anisotropic optical properties induced by the microstructures were systematically studied. The structure of prepared YF_3_ CTFs was characterized by X-ray diffraction (XRD). The surface and cross-sectional morphology of the YF_3_ CTFs were viewed by field-emission scanning electron microscopy (FE-SEM). Mueller matrix ellipsometry (MME), a powerful non-destructive and sensitive tool to study CTFs with high optical data precision, was employed to analyze the anisotropic optical properties of fabricated YF_3_ CTFs [24,25].

## 2. Theory and Optical Modeling

**Figure 1:**
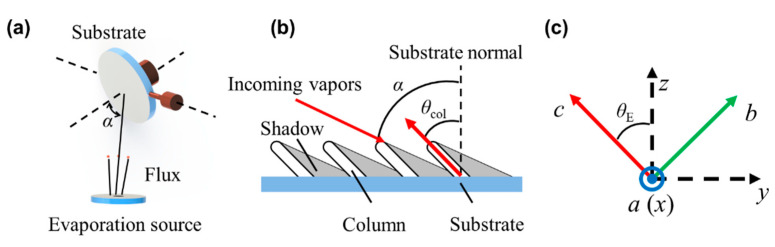
(**a**) Schematic diagram of the glancing angle deposition (GLAD) method for electron beam evaporation, (**b**) columnar thin film fabricated by the GLAD method, and (**c**) the laboratory coordinate system (*x*, *y*, *z*) and the principal coordinate system (*a*, *b*, *c*).

As shown in Figure 1a, the GLAD technique is applied to fabricate thin films on a substrate with an obliquely incident angle *α* of the vapor flux. The randomly deposited particles at the initial stage of deposition cause nuclei to be distributed over the surface, resulting in the ballistic shadowing of the surrounding regions. The limited surface mobility of adatoms prevents growth in the shadowed regions, thereby restricting further growth to the tops of the nuclei, which develop into tilted columnar structures [26]. Then, the isolated columns grow oriented toward the particle flow source, forming an inclined column angle *θ*_col_ between the substrate surface normal and the column direction, as shown in Figure 1b.

The highly oriented structure of the inclined columns induces optical anisotropy [6]. In this case, the orthogonal electric field components are coupled due to the columnar structure and the porosity. Thus, an MME measurement is required to fully characterize the polarization-dependent optical response of anisotropic films. In the MME measurement, the sample is represented by a 4 × 4 Mueller matrix M, describing the effect on the light with Stokes vector [27]

(1) $$S_{out}=M\;S_{in},$$

where S*_in_* and S*_out_* represent the Stokes vector of incident light and emerging light, respectively.

In the laboratory Cartesian coordinates (*x*, *y*, *z*), the columnar film is described by the second-rank tensor *ε*, written as

(2) $$\varepsilon =\left(\begin{array}{lll} \varepsilon_{xx} & \varepsilon_{xy} & \varepsilon_{xz}\\ \varepsilon_{yx} & \varepsilon_{yy} & \varepsilon_{yz}\\ \varepsilon_{zx} & \varepsilon_{zy} & \varepsilon_{zz} \end{array}\right).$$

In this coordinate system, the *y*-axis is parallel to the projection direction of columns on the substrate and the *z*-axis is parallel to the normal of film surface. For mathematical convenience, the dielectric tensor can be expressed by the principal dielectric constants through coordinate transformation. As shown in Figure 1c, the (*x*, *y*, *z*) system was transformed into the principal coordinate system (*a*, *b*, *c*) through two Euler rotations (*θ*_E_, *ϕ*). The inclination angle *θ*_E_, defined as the rotation from the *z*-axis to the *c*-axis, was considered to be equal to the inclined column angle *θ*_col_. The azimuth orientation *ϕ*, defined as the rotation around the *z*-axis, was set to zero when the columns were parallel to the plane of incidence (*y*-*z* plane). After coordinate transformation, the dielectric tensor in the (*a*, *b*, *c*) system was expressed as

(3) $$\varepsilon =\left(\begin{array}{lll} \varepsilon_{xx} & \varepsilon_{xy} & \varepsilon_{xz}\\ \varepsilon_{yx} & \varepsilon_{yy} & \varepsilon_{yz}\\ \varepsilon_{zx} & \varepsilon_{zy} & \varepsilon_{zz} \end{array}\right)=A^{T}\left(\begin{array}{lll} \varepsilon_{a} & 0 & 0\\ 0 & \varepsilon_{b} & 0\\ 0 & 0 & \varepsilon_{c} \end{array}\right)A.$$

## 3. Experimental Details

### 3.1. Materials and Sample Preparation

The YF_3_ CTFs were prepared by electron beam evaporation in a vacuum using the GLAD method. Single-side polished crystal <100> n-type silicon (99.99% in purity) wafers with a thickness of 350 ± 20 μm and a size of 10 mm × 10 mm were used as substrates. Granular YF_3_ (99.99% in purity) was heated by an electron beam at a high voltage of 7.8 kV and deposited onto the silicon substrates located 60 cm away from the evaporation source at a deposition rate of 0.20−0.25 nm s^−1^. The deposition angles were 0°, 66°, 70°, 78°, and 82°, respectively. All depositions were performed at a substrate temperature of 300 K and an operating pressure of 7 × 10^−5^ Torr.

### 3.2. Characterizations

The structural characteristics of the YF_3_ films were investigated by XRD with Cu-K*α* (*λ* = 0.154056 nm) radiation (Bruker D8 ADVANCE) in the scanning range of 10.0°−45.0° with a step of 0.02°. The surface morphology and the cross-section of the YF_3_ films were viewed by FE-SEM (Hitachi, S-4800 FE-SEM). The optical properties of the YF_3_ samples were characterized by a variable-angle Mueller matrix ellipsometer (RC2, J. A. Woollam), which worked in the reflection mode with a dual-rotating compensator configuration [24,28]. The 4 × 4 Mueller matrices were measured over a spectral range of 300−1650 nm (i.e., 4.1 to 0.75 eV) at two incident angles 65° and 75°, respectively. The azimuth of samples rotated from 0° to 360° at a 45° interval. The analysis was performed with the software CompleteEASE (J. A. Woollam) [28].

## 4. Results and Discussion

### 4.1. Microstructure of YF_3_ CTFs

**Figure 2:**
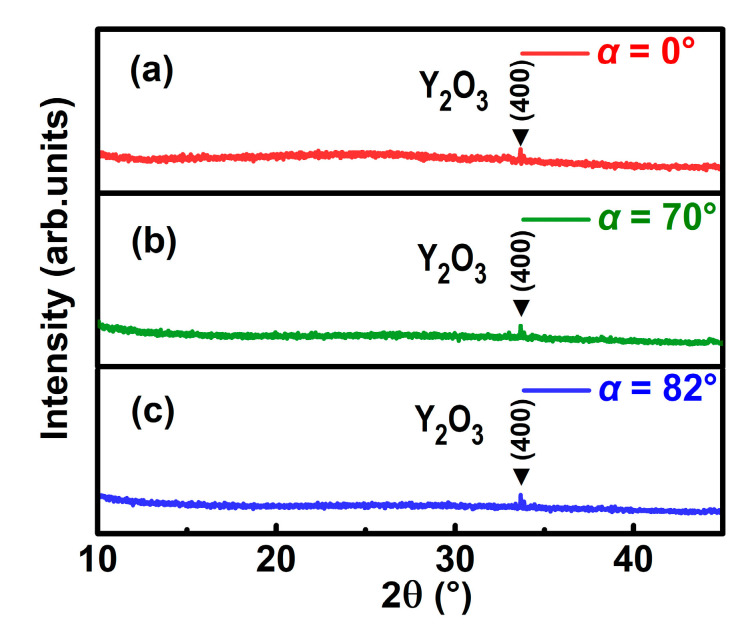
XRD patterns of yttrium fluoride (YF_3_) films deposited at the angles of 0° (**a**), 70° (**b**), and 82° (**c**).

Figure 2 presents the XRD patterns of as-deposited YF_3_ films grown on silicon substrates with deposition angles of 0°, 70°, and 82°, respectively. The (400) peak of cubic Y_2_O_3_ was observed at 2*θ* ≈ 33.7° in all the XRD spectra, indicating that Y_2_O_3_ was formed in all samples. No obvious diffraction peak corresponds to crystalline YF_3_, which means the intrinsic YF_3_ films are all amorphous. Since all YF_3_ samples were deposited at room temperature without annealing, this phenomenon can be attributed to the self-shadowing effect and limited adatom diffusion [6].

**Figure 3:**
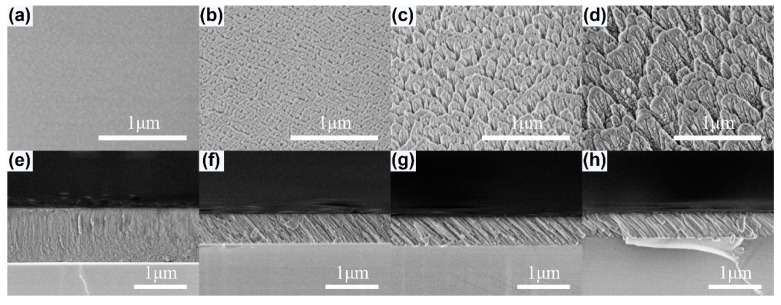
Top views (**a**–**d**) and cross-sectional views (**e**–**h**) of the FE-SEM images for YF_3_ films deposited at 0° (**a**,**e**), 70° (**b**,**f**), 78° (**c**,**g**), and 82° (**d**,**h**), respectively.

Figure 3a–d show the surface morphologies of the as-deposited YF_3_ films. The YF_3_ film grown at *α* = 0° is dense and shows a uniform morphology without voids. When *α* = 70°, the film becomes loose, with small pores appearing. In the case of *α* = 82°, the pores between columns become large in a rather loose film. The inclined columnar structures of the YF_3_ films are viewed in the cross-sectional SEM images (e–h) in Figure 3. The thickness *d* of YF_3_ films for each sample is 1104.6 nm (a, e), 560.2 nm (b, f), 546.5 nm (c, g), and 451.5 nm (d, h). The inclination angle *θ*_col_ of YF_3_ films observed from the SEM images is 0° (a, e), 29° (b, f), 38° (c, g), and 42° (d, h). As the value of *α* increases, the columns are increasingly separated and inclined due to the self-shadowing effect, which prevails over the surface diffusion of adatoms and results in porous and low-density films.

### 4.2. Optical Properties of YF_3_ CTFs

The optical properties of the prepared YF_3_ films were characterized by the MME. The dense YF_3_ films deposited at normal incidence of vapor flux (*α* = 0°) were characterized first to determine the dispersion function of YF_3_. The dispersion relations of the dense YF_3_ films were obtained by evaluating the 4 × 4 Mueller matrices [29,30]. The three-term Cauchy relation was used to describe the dispersion function of the YF_3_ films in the spectral range of 300−1650 nm, expressed as [31]

(4) $$n=A+\frac{B}{\lambda^{2}}+\frac{C}{\lambda^{4}},$$

where the refractive index *n* is a function of the wavelength *λ*, and the fitting parameters *A*, *B*, and *C* are material-dependent constants in the model. The optical constants of the dense YF_3_ films were not affected appreciably by their thickness, which is much thicker than the nucleation layer [32]. The constants for the dense YF_3_ film were obtained to be *A* = 1.477 ± 0.0005, *B* = (3.559 ± 0.005) × 10^3^ nm^2^, and *C* = 1.302 ± 0.005 nm^4^. The results are used as a baseline for comparing the YF_3_ CTFs deposited at the oblique incidence of vapor flux (*α* > 0°).

**Figure 4:**
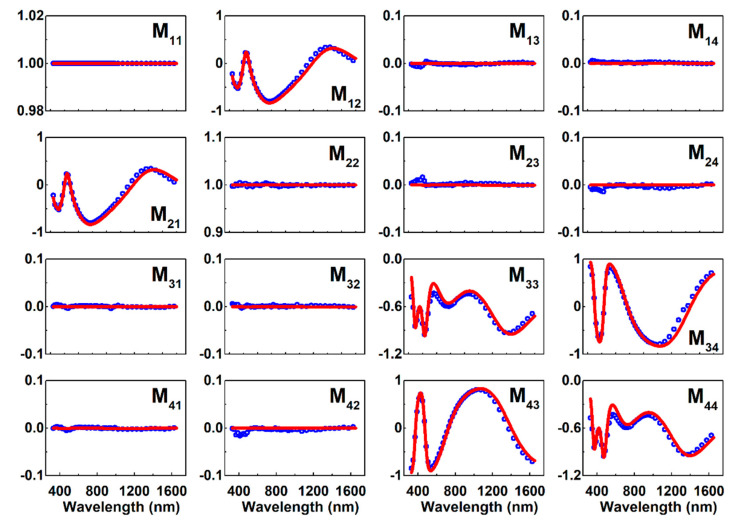
Measured (blue dots) and simulated (red lines) Mueller matrix elements normalized by the M_11_ for the YF_3_ sample prepared at a deposition angle *α* = 82° in the wavelength range of 300−1650 nm. The measurements were performed at a light incident angle *θ* = 65° and an azimuth orientation *ϕ* = 0°.

The YF_3_ CTFs deposited at *α* > 0° were characterized subsequently. The individual columns in the YF_3_ CTFs were assumed to satisfy the same dispersion functions as the dense YF_3_ film deposited at *α* = 0°. Figure 4 shows the experimental and the fitted Mueller matrix of the sample grown at *α* = 82° over the spectral range of 300−1650 nm, normalized by the M_11_. To interpret the MME data for the YF_3_ CTFs, structural parameters, i.e., the thickness *d* and the inclination angle *θ*_col_, are required to link to the Mueller matrix data through an appropriate optical model. A biaxial orthorhombic model, similar to that reported by Gospodyn et al., was selected to model the optical properties of the YF_3_ CTFs [32]. During the fitting process, the structure parameters *d* and *θ*_col_ do not change with light wavelength, incident angle, and azimuth orientation.

The Bruggeman effective-medium approximation (EMA) was employed for the YF_3_ CTFs to evaluate the Mueller matrix data. The mixed medium was assumed to be composed of film material and void material [33]. The void material was assumed to be a medium with a refractive index of 1 and an extinction coefficient of 0 [32,33]. Since the CTF is a biaxial anisotropic medium, the direction-dependent Bruggeman EMA was applied accordingly. In this model, the volume fraction of YF_3_ was constrained to be constant in all directions for a certain point in the film. Thus, the effective dielectric constants along the principal axis are expressed as [34]

(5) $$\left(1-g_{j}\right)\varepsilon_{j}^{2}+B_{j}\varepsilon_{j}-g_{j}\varepsilon_{\mathrm{YF}3}=0,\;\left(j=a,b,c\right),$$

where *B_j_* is given by

(6) $$B_{j}=\left(g_{j}-f\right)\varepsilon_{\mathrm{YF}3}-\left(1-f-g_{j}\right),$$

where *f* represents the volume fraction of YF_3_ in the film, and *g_j_* represents the depolarization factor for the optical *j* axis [35]. The void fraction *f*_void_ is equal to 1 − *f*. The depolarization factors depend on the shape of the columns, with *g_a_* + *g_b_* + *g_c_* = 1. With the direction-dependent Bruggeman EMA, the effective principal dielectric constants *ε_j_* are matched to the values obtained from the biaxial orthorhombic model by varying the fitting parameters *g_a_*, *g_b_*, *f*, and the bulk-like dielectric function *ε*_YF3_.

**Figure 5:**
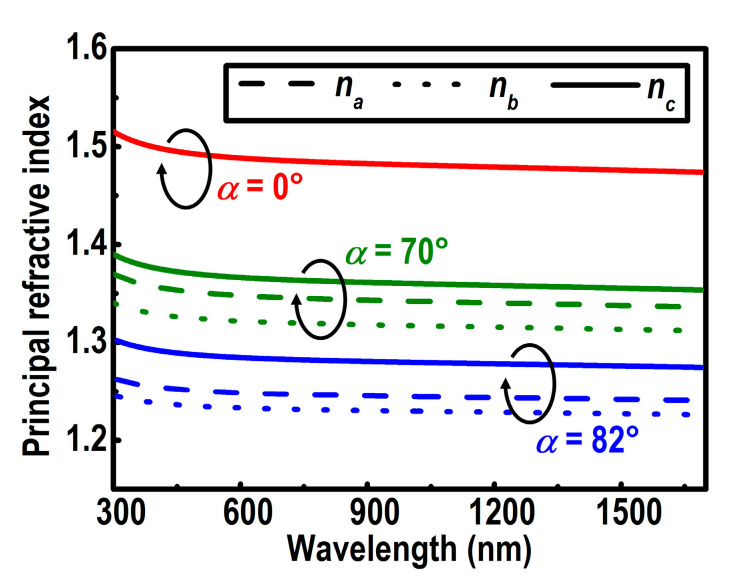
Principal refractive indices *n_a_* (dashed lines), *n_b_* (dotted lines), and *n_c_* (solid lines) of the biaxial YF_3_ columnar thin films (CTFs) at deposition angles of 0°, 70°, and 82°, respectively.

The fitted refractive indices are depicted in Figure 5. All the three principal indices of refraction for the YF_3_ CTFs decrease slightly as the wavelength increases from 300 nm to 1650 nm. The value of *n_c_* is noted to be the highest of the three principal refractive indices. For example, *n_a_* = 1.249, *n_b_* = 1.233, and *n_c_* = 1.286 are acquired for the 82°-deposited film at 550nm. Since the structure in the column direction can be regarded as laminar with the electric field parallel to the material layer, the measured depolarization factor along this axis is *g_c_* ≈ 0 [36].

**Figure 6:**
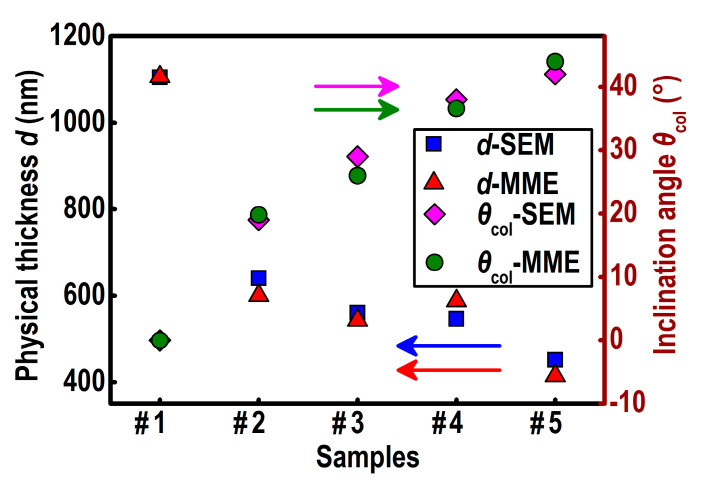
Physical thickness *d* and inclination angle *θ*_col_ of YF_3_ films measured by SEM and Mueller matrix ellipsometry (MME).

The thickness *d* and the inclination angle *θ*_col_ of YF_3_ films obtained from the SEM and the MME are shown in Figure 6. The MME results are consistent with the SEM results for two parameters. The difference between the two measurement results can be attributed to the fact that the SEM measurement was performed in the micrometer range while the MME measurement was performed in the millimeter range.

**Figure 7:**
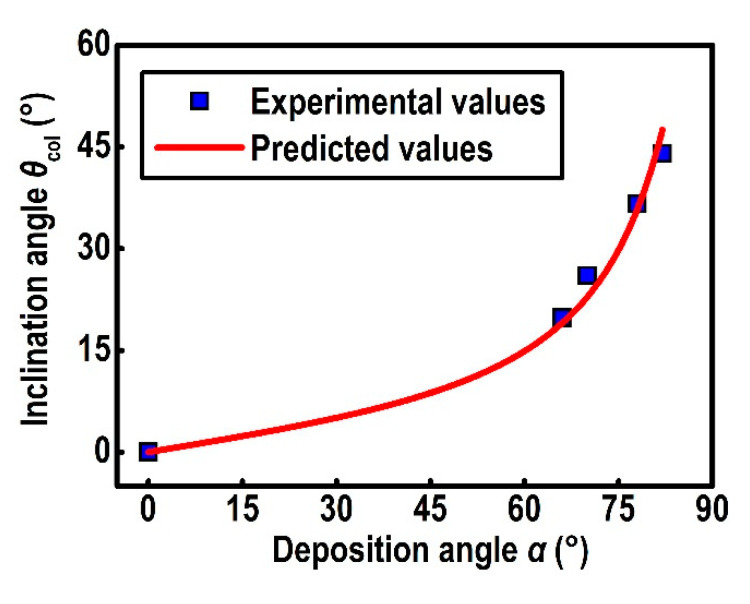
The inclination angle for YF_3_ CTFs versus the deposition angle. The filled squares indicate the experimental values, and the solid curve indicates the predicted values by the modified tangent-rule equation.

The relation between the deposition angle and the inclination angle fitted from MME is shown in Figure 7. The experimental values were fitted by the modified tangent-rule equation [37]

(7) $$\theta_{\mathrm{col}}=\tan^{-1}\left(E\tan\alpha \right),$$

where the optimized value of the constant *E* in the equation is 0.153.

**Figure 8:**
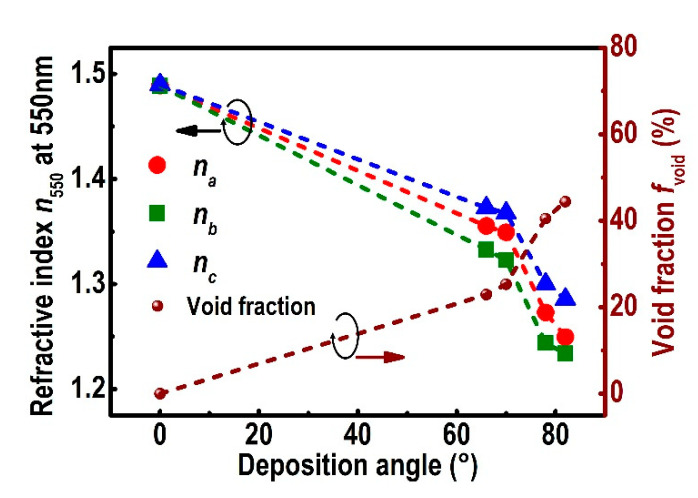
Void fractions and principal refractive indices *n_a_* (red dots), *n_b_* (green squares), and *n_c_* (blue triangles) at 550 nm for the YF_3_ columnar films grown at various deposition angles. Dashed lines are guides for the eye.

The principal refractive indices for YF_3_ films versus the deposition angle at 550 nm are shown in Figure 8. All the principal refractive indices decrease as the deposition angle increases from 66° to 82°. The refractive index perpendicular to the column direction (*n_b_*) for the sample deposited at *α* = 82° is approximately 1.233 at 550 nm, which is much lower than that of dense YF_3_ films (1.489 at 550 nm). According to the two-dimensional simulation predictions, the decrease in the refractive index of YF_3_ CTFs is caused by the decrease in the volume fraction of YF_3_ with the increase in the deposition angle *α* [38]. These results are consistent with studies on niobium pentoxide, magnesium fluoride, and tungsten oxide [12,32,39].

Figure 8 shows the void fraction *f*_void_ calculated with the Bruggeman EMA versus the deposition angle. The void fraction of YF_3_ films increases with the increase in the deposition angle *α*. At *α* = 82°, the void fraction increases to approximately 45%, which implies that nearly half of the YF_3_ CTF is filled with voids. The high void fraction is the result of more separated and inclined columns due to the enhanced atomic self-shadowing effect and limited adatom diffusion [6]. By adjusting the angle of particle flux, the effective refractive index and the void fraction of YF_3_ CTF can be designed within a continuous range of values.

**Figure 9:**
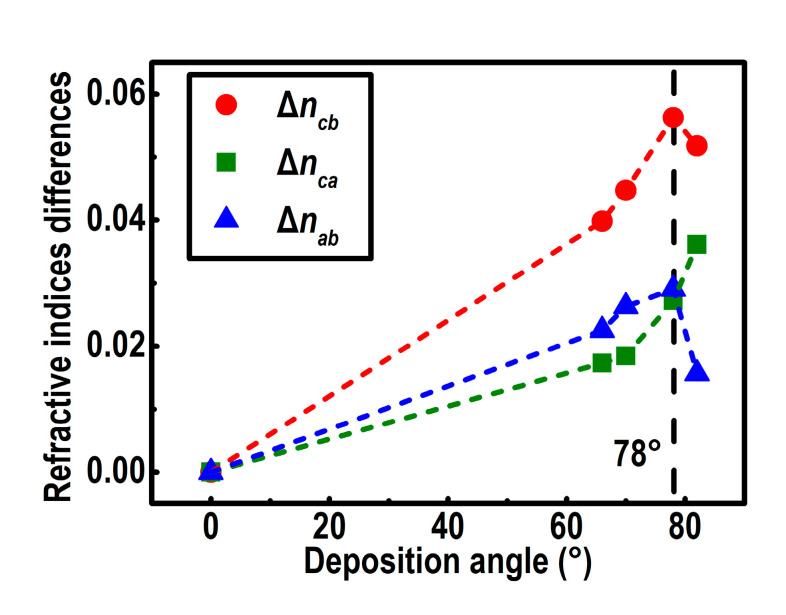
Refractive indices differences Δ*n_cb_* (red dots), Δ*n_ca_* (green squares), and Δ*n_ab_* (blue triangles) at 550 nm for YF_3_ films versus deposition angle. Dashed lines are guides for the eye.

The highly oriented nanostructure of inclined columns indicates that the YF_3_ CTFs are biaxially anisotropic, with the long axis along the column direction [6]. The differences in the three principal indices of refraction, Δ*n_cb_*, Δ*n_ca_*, and Δ*n_ab_*, are used to quantify its anisotropy, which is defined by the absolute values of *n_c_* − *n_b_*, *n_c_* − *n_a_*, and *n_a_* − *n_b_*, respectively. Figure 9 illustrates the refractive indices differences versus deposition angles at *λ* = 550 nm. At *α* = 78°, Δ*n_cb_* and Δ*n_ab_* reach the maximum 0.056 and 0.029, respectively. A larger deposition angle leads to an increase in Δ*n_ca_*, along with a decrease in Δ*n_cb_* and Δ*n_ab_*. The differences in the three principal refractive indices of the YF_3_ CTFs show a strong dependence on the deposition angle. A smaller deposition angle results in a smaller structural anisotropy of the film, as well as a minor optical anisotropy. However, a too oblique deposition angle will result in a low volume fraction of YF_3_ and a low effective refractive index of the film. The volume fraction of YF_3_ is another main factor affecting the difference in refractive index. The critical volume fraction of YF_3_ of the maximum refractive index differences is deduced to be 59.5% from Figure 8. The optimal deposition angle should balance these two competing factors to yield the maximum differences in the three principal refractive indices [13]. In addition, according to the results of the XRD measurements, the intrinsic YF_3_ is amorphous, which means the optical anisotropy caused by crystallization is negligible.

In practical application, the GLAD technique provides a solution for preparing a gradient- refractive-index structure, which can be used for anti-reflectors, solar cell absorbers, and radiative coolers [8,40]. The anisotropic structure of films prepared by the GLAD technique will also induce anisotropy in the thermal, electrical, and magnetic properties of thin films [11,25,41].

## 5. Conclusions

A series of YF_3_ CTFs with various column angles were fabricated by the GLAD method. The as-deposited YF_3_ films grown at different deposition angles were found to be amorphous. The columns of YF_3_ CTFs became increasingly separated and inclined as the deposition angle increased. The structural parameters obtained from the MME agree with those viewed from the FE-SEM images for inclination angle and physical thickness. The optical properties of the studied samples obtained from the MME measurement show that the highly oriented YF_3_ CTFs were biaxially anisotropic, with the highest refractive index along the column direction. The three principal refractive indices and the volume fraction of YF_3_ evaluated from the direction-dependent Bruggeman EMA decreased significantly as the deposition angle increased due to the self-shadowing effect and limited adatom diffusion. In addition, the refractive index differences of the columnar thin films in the three principal directions strongly depend on the deposition angle and the volume fraction of YF_3_.
